# Retention of Halogenated Solutes on Stationary Phases Containing Heavy Atoms

**DOI:** 10.3390/molecules18055163

**Published:** 2013-05-06

**Authors:** Toshio Miwa, Atsushi Yamamoto, Mitsuru Saito, Yoshinori Inoue

**Affiliations:** 1 College of Bioscience and Biotechnology, Chubu University, 1200 Matsumotocho, Kasugai-shi, Aichi 487-8501, Japan; E-Mail: akmiy@isc.chubu.ac.jp; 2 Nippon Filcon Co., Ltd., 2220 Ohmaru, Inagi-shi, Tokyo 206-8577, Japan; E-Mails: m-saito@filcon.co.jp (M.S.); y-inoue@filcon.co.jp (Y.I.)

**Keywords:** solid-phase extraction, heavy atom effect, halophenoxy functional groups, induced dipole moment

## Abstract

To examine the effects of weak intermolecular interactions on solid-phase extraction (SPE) and chromatographic separation, we synthesized some novel stationary phases with a heavy atom effect layer by immobilizing halogenated aromatic rings and hydroxyl groups onto the surface of a hydrophilic base polymer. Using SPE cartridges packed with the functionalized materials, we found that the heavy atom stationary phases could selectively retain halophenols in organic solvents, such as 1-propanol which blocks the hydrogen bonding, or acetonitrile which blocks the π-π interaction. The extraction efficiency of the materials toward the halophenols depended on the dipole moments of phenoxy groups present as functional groups. On the other hand, the extraction efficiency of solutes toward the functional group depended on their molar refractions, *i.e.*, induced dipole moments. The retention of the solutes to the stationary phase ultimately depended on not only strong intermolecular interactions, but also the effects of weak interactions such as the dispersion force.

## 1. Introduction

The basic concept of solid-phase extraction (SPE) was established in the 1960s, and the development and use of SPE expanded tremendously during the 1980s. SPE overcomes many of the drawbacks of liquid-liquid extraction and has become an alternative to conventional sampling and pre-concentration methods for instrumental analysis. The principles and instruments of SPE have been reviewed [1,2,3,4,5]. Classical adsorbents for SPE, such as C_18_ and styrene-divinylbenzene copolymers, can pre-concentrate a wide variety of hydrophobic solutes in aqueous samples. However, these adsorbents are not highly selective—they tend to retain other matrix compounds that can interfere with the desired analysis.

Two types of stationary phases have been used to recognize specific structures. One contains molecularly imprinted polymers (MIP) [6,7] and the other contains antibodies [8]. Two major approaches have been carried out in order to recognize the differences between delicate structures involved in the production of MIPs or antibodies. However, both of these stationary phases have some drawbacks. A problem with MIP-based SPE, which employs the analyte as an imprint molecule, is that complete removal of the template is difficult and the slow release of the residual template leads to false positive results. Synthesis of MIPs that minimize these problems is time consuming and expensive. The resulting adsorbents may still show selectivity for compounds other than the target analyte. On the other hand, antibodies used for the stationary phase have high target selectivity, but they are difficult to store and are not stable in some organic solvents. Aptamers, which are synthetic nucleic acids with selective binding properties, have been used in analytical chemistry [9,10], but their use in SPE has just begun.

Multifunctional adsorbents, which contain two or more functional groups, have chemical affinities that are different from the chemical affinities of MIPs. However, such adsorbents which combine electrostatic and hydrophobic interactions are lacking in recognition of target molecules [11]. Consequently, we examined the use of weaker intermolecular interactions such as nonbonding intermolecular forces to selectively retain the target analye. First, we used a multifunctional adsorbent that combined a weaker force with a strong intermolecular interaction. Next, we used a solvent that blocks the strong force to release the matrix compounds. The multifunctional adsorbent retains only compounds whose binding is governed by both strong and weak intermolecular interactions. With this approach adsorbents that can retain the hydrophilic solutes in the organic solvent and the ionic solutes in the electrolyte might be developed. Tanaka and co-workers reported that a pentabromobenzyl-bonded silica column strongly retained solutes based on the dispersion force [12], and that a *p*-nitro-phenyl-bonded column preferentially retained solutes based on the dipole-dipole interaction [13]. These interactions helped to retain solutes on a conventional reversed-phase HPLC column [14]. Here, we attempted to design new stationary phases for SPE by immobilizing halogenated phenoxy groups on a hydrophilic polymer surface. The selectivity of these adsorbents for halogenated organics was found to depend on dispersion forces.

## 2. Results and Discussion

### 2.1. Functional Groups Contents

We thought that it was necessary to reduce the influence of the base gel as much as possible to clarify the interaction of the halogenated phenoxy groups. Silica gel suffers from the influence of silanol groups and residual metals, and styrene polymers display strong hydrophobic effects. Consequently the functional groups were introduced onto a hydrophilic metacrylate base gel.

According to the elemental analysis results in Table 1, the phenoxy groups contents were not significantly different among the brominated adsorbents. However, more phenoxy groups were introduced in the 2,4-dichlorophenoxy (DCP) adsorbent than in brominated adsorbents. Moreover, the phenoxy group content in phenoxy (Phe) adsorbent was two times larger than that in DCP. It seemed that the reactivity is influenced by the acid dissociation constant and steric hindrance around the phenoxy group and molar refraction of phenols. In the following experiments, the SPE efficiency of phenolic solutes was evaluated with the same amount of the synthesized adsorbents independently of the contents of functional groups.

**Table 1:** Contents of functional group in the prepared adsorbents.

Adsorbents	Contents of phenoxy groups/mmol g^−1^
Pentabromophenoxy (PBP)	0.30
2,4,6-Tribromophenoxy (TBP)	0.23
2,4-Dibromophenoxy (DBP)	0.34
4-Bromophenoxy (4BP)	0.43
2,4-Dichlorophenoxy (DCP)	0.56
Phenoxy (Phe)	1.22

### 2.2. SPE Efficiencies of the Synthesized Adsorbents

Although benzenes in methanol/water (5+5) were retained by every adsorbent, their extraction efficiencies varied widely, ranging from about 30% on Diol to 100% on HLB, but benzenes in organic solvents passed through the cartridges with being retained by any adsorbent. Judging from these results, the adsorption behavior of benzenes in all adsorbents used here was based on a hydrophobic interaction, and the adsorbents did not develop any selectivity for benzenes. On the contrary, phenols interact effectively with halogenated phenolic groups. Phenols in water were retained perfectly by every adsorbent, and phenols in *n*-hexane were also retained by all adsorbents except for C18. The order of solvents on the extraction efficiency of phenols was water ≥ *n*-hexane » ethyl acetate > acetone ≥ acetonitrile > 1-propanol > methanol.

**Figure 1:**
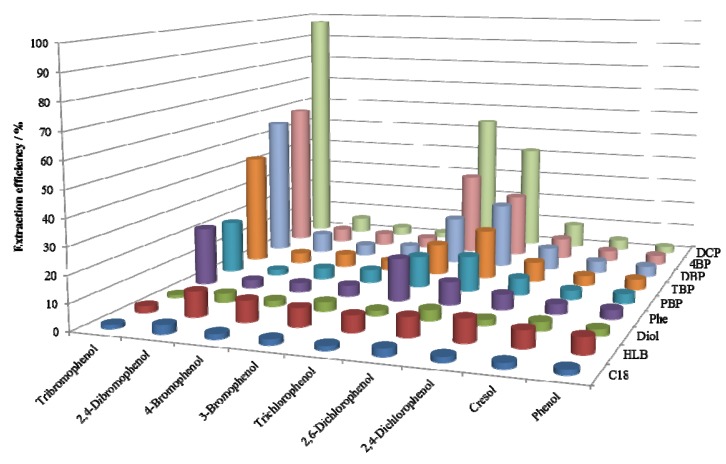
Extraction efficiencies (n = 2) of phenols dissolved in 1-propanol on various adsorbents.

The extraction efficiencies of phenols in 1-propanol are presented in Figure 1. Since 1-propanol has high solvent power, the adsorbents cannot be expected to retain solutes by the hydrophobic effect when 1-propanol is used as the solvent. Moreover, the hydroxyl group in this molecule would weaken the formation of hydrogen bonds between the hydrophilic moieties in the stationary phase and the solutes. For the reasons mentioned above, phenols in 1-propanol were not retained at all on reversed-phase adsorbents such as C18 and HLB, but the extraction efficiencies of 2,4,6-tribromophenol in 1-propanol on 4-bromophenoxy (4BP) and 2,4-dibromophenoxy (DBP) adsorbents reached about 60%, and the extraction efficiency on DCP adsorbent was the highest of all. The hydroxyl groups seemed to play an important role in the retention of solutes because these adsorbents had almost no affinity for halobenzenes.

**Figure 2:**
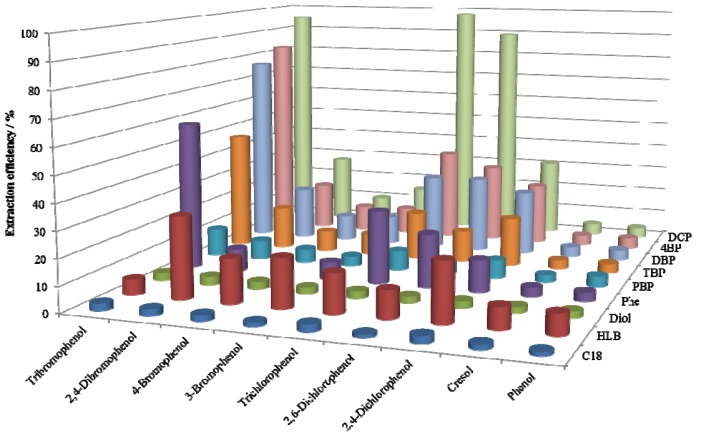
Extraction efficiencies (n = 2) of phenols dissolved in acetonitrile on various adsorbents.

The extraction efficiencies of phenols in acetonitrile are presented in Figure 2. Acetonitrile also has high solvent power and its nitrogen atom takes part in hydrogen bonds. Moreover, the π electron in the molecule would weaken the π-π interaction between the aromatic rings on the stationary phase and the solutes. Thus, it was thought that the intermolecular forces between the stationary phase and phenols in acetonitrile were weaker than those in 1-propanol. However, the extraction efficiencies of the adsorbents were inconsistent with this assumption. It was assumed that the retentions of halophenols by the halogenated phenoxy adsorbents were mainly induced by hydrogen bonds, and the selectivity for halophenols was governed by the dispersion force of heavy atoms.

### 2.3. Effect of Orientation Dipole Moments of Functional Groups on the Retention

According to Figure 1, Figure 2, the order of the affinity of functional groups to halophenols was DCP > 4BP ≅ DBP > 2,4,6-tribromophenoxy (TBP) ≅ Phe > pentabromophenoxy (PBP). However, considering the contents of functional groups in Table 1, the proper order should be DCP ≥ 4BP ≅ DBP > TBP > PBP ≥ Phe due to the extremely high content of phenoxy groups.

Free rotation of phenol moieties in the stationary phase is obstructed because they are fixed on the base polymer, but the orientation dipole moment remains. The order of permanent dipole moments of phenols (Table 2) introduced as functional groups agrees approximately with the affinity mentioned above. Consequently, the affinity was considered to be derived from the dipole moments of the functional groups.

**Table 2:** Dipole moment data.

Phenols	Dipole moment/D	References
2,4-Dichlorophenol	3.019 *	[15]
2,4-Dibromophenol	2.64–2.99 *	[16,17]
4-Bromophenol	2.15–2.78	[18,19]
2,4,6-Tribromophenol	1.44–2.15	[18,20]
Pentabromophenol	1.73	[16]
Phenol	1.22–1.86	[18,21]

* OH group does not take part in intramolecular hydrogen bonding with the *ortho*-substituted halogen.

### 2.4. Effect of Induced Dipole Moments of Solutes on Retention

Based on the data shown in Figure 1, Figure 2, the order of the affinity of phenols on DCP adsorbent was tribromo > trichloro > 2,6-dichloro > 2,4-dichloro ≥ 2,4-dibromo > 3-bromo > 4-bromo » 4-methyl > H.

Phenolic solutes can rotate freely and their time-averaged dipole moments are zero. Polar solute molecules drawn toward the functional group by hydrogen bonding have an orientational polarizability arising from the dipole moment of the phenoxy functional group. The orientational polarizability defined by the strength of the induced dipole moment is hard to measure, so the molar refraction, R_D_, was calculated from the following equation:

R_D_ = (n^2^ − 1)*M* (n^2^ + 2)^−1^ρ^−1^ (1)

where n is the refractive index, *M* is the molecular weight, and ρ is the mass density. Molar refraction is governed by the polarization of the molecule, and increases in proportion to the polarizability. The calculated molar refractions are given in Table 3. Although the molar refractions of disubstituted halophenols calculated by using the predicted values of the refractive index cannot be directly compared to the measured values, it is clear that the order of the affinity mentioned above almost agrees with the molar refractivity but not with the dipole moment. For example, solutes with low molar refractivity such as phenol and cresol were hardly retained by DCP adsorbent, and monosubstituted halophenols also showed weak affinity. The dipole-induced dipole potential energy, V, is given by:

V = −4μ^2^α (4πε_0_)^−2^*r*^−6^ (2)

where μ is the dipole moment of the functional group, α is the polarizability of the solute, ε_0_ is the dielectric permittivity of free space, and *r* is the distance between the center of the functional group and the solute. The potential energy determining the retention is the product of the square of the dipole moment of the functional group and the polarizability of the solute. However, this equation cannot fully explain all the factors affecting retention. For example, the steric hindrance around the methyl group in cresol would weaken the π-π stacking interaction. The solute molecule should be as close to the functional group as possible to develop strong affinity because the potential energy is inversely proportional to the sixth power of the distance between the two molecules. It was found that the weak intermolecular forces developed very strong extraction efficiency in combination with the strong long-range interactions such as hydrogen bonding.

**Table 3:** Calculation of molar refraction for halophenols.

Phenols	Dipole moment/D	Refractive index	Specific volume/g cm^−^^3^	Molar weight/g mol^−^^1^	Molar refraction/cm^3^ mol^−^^1^
2,4,6-Tribromophenol	1.45–2.15	1.7 ^a^	2.55 (20 °C)	330.8	50.1 (51.2 ^b^)
2,4,6-Trichlorophenol	1.38–2.00 [18,20]	1.6 ^a^	1.675	197.45	40.3 (42.8 ^b^)
2,6-Dichlorophenol	1.77–2.15 [15,21]	1.594 ^b^	1.532	163.0	36.1 (37.9 ^b^)
2,4-Dichlorophenol	0.846–3.019	1.594 ^b^	1.383	163.0	40.0 (37.9 ^b^)
2,4-Dibromophenol	0.37–2.99	1.643 ^b^	2.07 (20 °C)	251.9	44.0 (43.5 ^b^)
3-Bromophenol	0.90–3.10 [16]	1.5957	1.63	173.0	36.2 (35.8 ^b^)
4-Bromophenol	2.15–2.78	1.5875	1.65	173.0	35.3 (35.8 ^b^)
4-Cresol	1.44–1.83 [18]	1.5312	1.0347	108.1	33.1 (33.0 ^b^)
Phenol	1.22–1.86	1.5425	1.07	94.1	27.7 (28.1 ^b^)

^a^ Values speculated using the data from ref. [18]; ^b^ Predicted values generated using the ACD/Labs’ ACD/PhysChem Suite.

## 3. Experimental

### 3.1. Reagents and Chemicals

Most reagents for the synthesis of phenoxy adsorbents were purchased from Wako Pure Chemical Industries (Osaka, Japan). Glycidylmethacrylate (GMA) and ethyleneglycol dimethacrylate (EGDM) (both from Tokyo Chemical Industries, Tokyo, Japan) were used as a functional monomer and a cross-linker, respectively. Butyl acetate and 3-methylbutanol were chosen as inert diluents for pore generation. As a polymerization initiator, 2,2-azobis(isobutyronitrile) was utilized. Polyvinylalcohol (n = 500) was used as a suspension stabilizer. As the precursor of the organic moieties in each stationary phase, Phe, 4BP, DBP and PBP were also purchased from Wako Pure Chemical Industries. Trimethylphenol, trichlorophenol, dichlorobenzene, dichlorophenol and xylene used as the analytes for the evaluation of extraction and retention properties were purchased from Tokyo Chemical Industries. Other analytes were purchased from Wako Pure Chemical Industries.

### 3.2. Synthesis and Characterization of the Adsorbents with Heavy Atom Effect

**Scheme 1:**
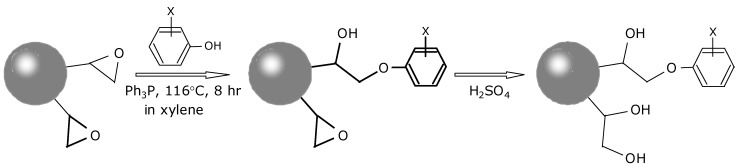
Synthesis steps used for preparing the phenoxy adsorbents.

A base resin for SPE adsorbent was prepared by a suspension copolymerization of GMA and EGDM according to our previous paper [22]. Its specific surface area was about 160 m^2^ g^−1^. Each phenol, *i.e.*, Phe, 4BP, DBP, TBP, PBP, and DCP, was introduced onto the base resin classified as 45–90 μm by heating in xylene at 116 °C for 8 h in the presence of triphenylphosphine catalyst. Residual epoxy groups were opened to diol groups by sulfuric acid. The synthesis procedure for the phenoxy adsorbents is shown in Scheme 1. The obtained resins were washed with excess acetone and dried. Diol resin was synthesized by treatment of the base resin with sulfuric acid to cleave the epoxy groups. An Oasis HLB from Waters (Milford, MA, USA) and an InertSep C18 from GL Sciences (Tokyo, Japan) were used for comparison with the prepared adsorbents in this experiment.

Elemental analysis of synthesized adsorbents was performed by combustion-ion chromatography (IC) using a Mitsubishi Chemical Analytech (Chigasaki, Kanagawa, Japan) AQF-100 combustion preparation station was combined with Metrohm Japan (Tokyo, Japan) DX-120 IC. Thermal oxidative digestion of 0.1 g halogenated phenoxy adsorbents were performed on the following conditions: oxygen 400 mL min^−^^1^, argon 350 mL min^−^^1^, water 0.05 mL min^−^^1^, temperature 900 °C. Halogen contents were measured by IC under the following conditions: column: Metrohm 12A (15 cm × 4.6 mm i.d.); eluent, 2.7 mM Na_2_CO_3_/0.3 mM NaHCO_3_; flow-rate, 1.3 mL min^−1^; temperature, 40 °C; detector, suppressed conductivity.

The functional group contents in the phenoxy adsorbents was calculated from the IR data (JASCO FT/IR-660Plus, JASCO, Tokyo, Japan) recorded in KBr pellets. The calibration curve was obtained by plotting the relative ratio of the out-of-plane bending absorption signal around 700 cm^−1^ for aromatic C-H to the carbonyl group stretching signal around 1,800 cm^−1^ *versus* the amount of anisole impregnated into the base resin.

### 3.3. Evaluation of the SPE Adsorbents

One hundred mg of each phenoxy adsorbent—4BP, DBP, TBP, PBP, DCP and TCP—and each reference adsorbent—Phe, Diol, HLB and C18—was packed in a 1 mL SPE cartridge (BondElut Reservoir, Varian, CA, USA). The cartridge was conditioned by passing 10 mL of methanol, 10 mL of acetone and 10 mL of same solvent as the analyte was loadedin, in that order. Benzenes and phenols as analytes were each dissolved in eight kinds of solvents, *i.e.*, *n*-hexane, ethyl acetate, acetone, acetonitrile, 1-propanol, methanol, methanol/water (5+5), and water. Each 10 mL aliquot of 10 mg L^−1^ of sample solution was loaded into the SPE cartridge. The solute concentration in the solution passed through the cartridge was measured by HPLC. Extraction efficiency is defined by (C_load_ − C_effluent_)/C_load_, where C_load_ is the solute concentration in the loaded solution and C_effluent_ is that in the effluent.

The LC system consisted of a GL sciences GL-7410 pump, a GL-7420 auto-sampler, a GL-7432 column oven and a GL-7452 photodiode-array detector (GL Sciences, Tokyo, Japan). The chromatographic separation was performed on a 15 cm × 4.6 mm i.d. reversed-phase column, InertSustain C18 (GL Sciences, 5 µm), maintained at 50 °C. A mobile phase consisted of water and acetonitrile was delivered at 1.5 mL min^−^^1^.

## 4. Conclusions

New adsorbents were synthesized in which various phenoxy groups were introduced onto the surface of hydrophilic base gels. The resulting materials had many halogen atoms in the functional groups and showed typical dispersion force retention features. Their strong affinity for halophenols seemed to be derived mainly from hydrogen bonding and later additional interactions of weak π-π stacking and weak dispersion forces. Consequently, the adsorbents had almost no retention for halobenzenes that were unable to form hydrogen bonds.

The affinity of the proposed adsorbents toward halophenols was explained by the dipole-induced dipole interaction, in which the affinity depends on the polarizability of the target solute. Adsorbents with a dipole moment appeared to be better at retaining polarizable solutes.

These findings clearly show that very weak physical forces such as the van der Waals force can be effectively used for SPE. Using combinations of functional groups with various physical forces should lead to the development of adsorbents with new properties and better SPE techniques.
